# Identification and characterization of a unique role for EDB fibronectin in phagocytosis

**DOI:** 10.1007/s00109-015-1373-0

**Published:** 2015-12-05

**Authors:** Sabrina Kraft, Verena Klemis, Carla Sens, Thorsten Lenhard, Christian Jacobi, Yvonne Samstag, Guido Wabnitz, Michael Kirschfink, Reinhard Wallich, G. Maria Hänsch, Inaam A. Nakchbandi

**Affiliations:** Max-Planck Institute of Biochemistry, 82152 Martinsried, Germany; Institute of Immunology, University of Heidelberg, 69120 Heidelberg, Germany; Department of Neurology, University of Heidelberg, 69120 Heidelberg, Germany; Department of Neurology, Krankenhaus Nordwest, 60488 Frankfurt, Germany

**Keywords:** Innate immunity, Bacterial infection, Phagocytosis, Fibronectin, EDB, EIIIB, αvβ3 integrin, Circulation, Plasma fibronectin

## Abstract

**Abstract:**

Plasma fibronectin is a circulating protein that facilitates phagocytosis by connecting bacteria to immune cells. A fibronectin isoform, which includes a sequence of 90 AA called extra-domain B (EDB), is synthesized de novo at the messenger RNA (mRNA) level in immune cells, but the reason for its expression remains elusive. We detected an 80-fold increase in EDB-containing fibronectin in the cerebrospinal fluid of patients with bacterial meningitis that was most pronounced in staphylococcal infections. A role for this isoform in phagocytosis was further suggested by enhanced EDB fibronectin release after internalization of *Staphylococcus aureus* in vitro. Using transgenic mouse models, we established that immune cell production of fibronectin contributes to phagocytosis, more so than circulating plasma fibronectin, and that accentuated release of EDB-containing fibronectin by immune cells improved phagocytosis. In line with this, administration of EDB fibronectin enhanced in vitro phagocytosis to a larger extent than plasma fibronectin. This enhancement was mediated by αvβ3 integrin as shown using inhibitors or cells from β3 integrin knockout mice. Thus, we identified both a novel function for EDB fibronectin in augmenting phagocytosis over circulating plasma fibronectin, as well as the mediating receptor. Our data also establish for the first time, a direct role for β3 integrin in bacterial phagocytosis in mammals.

**Key messages:**

• Fibronectin containing an extra domain called EDB is released in bacterial meningitis.

• EDB-containing fibronectin enhances phagocytosis more than plasma fibronectin.

• The enhancement is mediated by activation of αvβ3 integrin in the presence of EDB.

**Electronic supplementary material:**

The online version of this article (doi:10.1007/s00109-015-1373-0) contains supplementary material, which is available to authorized users.

## Introduction

Fibronectin is an extracellular matrix protein that is produced by almost all mammalian cells [1]. It affects proliferation, migration, differentiation, and survival [2, 3]. These different functions are made possible by both the presence of several isoforms and the binding to a variety of integrins. Two of the isoforms of fibronectin are defined by the presence of extra domains called extra domain A (EDA) and extra domain B (EDB). Even though most of the studies were performed with the circulating isoform of fibronectin, which lacks both EDA and EDB and is called plasma fibronectin (pFN), studies using isoform-specific knockouts lacking either EDA, EDB, or both domains found that these two isoforms contribute to vasculogenesis in embryos and angiogenesis in cancer [4–7]. In addition, EDA-containing fibronectin plays a role in a variety of pathologic entities such as liver fibrosis and diabetes mellitus, but no further functions of EDB-containing fibronectin have been characterized [8, 9]. Furthermore, while a specific binding site to integrins was characterized for EDA, no receptor for EDB has been identified in vivo yet [1, 10].

Studies on the circulating isoform of fibronectin (plasma fibronectin) have shown that fibronectin facilitates adherence of bacteria to other cells and thus acts as an adhesion molecule on mammalian cells [11]. Strains of *Staphylococcus aureus* for example express several molecules such as fibronectin-binding proteins (Fnbp) that enable bacteria to attach to and invade tissues [12, 13]. The most widely known function of fibronectin in phagocytosis is as a bridge between the bacteria and integrin α5β1, the classical fibronectin receptor [14, 15]. Even though fibronectin was originally shown to act as an opsonin by marking the bacteria and enhancing phagocytosis [16, 17], experimental data also show that fibronectin enhances phagocytosis irrespective of whether it binds to bacteria or not [17]. Neither EDA- nor EDB-containing fibronectin was studied in the context of phagocytosis.

Phagocytosis is evolutionally critical and beneficial. Therefore, much overlap in the stimulators and enhancers of phagocytosis exists, and several integrins are involved in phagocytosis. The only β2 subunit-containing integrin involved in phagocytosis is α_M_β2 integrin (also called complement receptor 3 or CD11b/CD18) which affects complement-activated phagocytosis of several pathogens including lipopolysaccharide-expressing bacteria [18–20]. Therefore, upregulation of β2 enhances phagocytosis [18]. Another mechanism of phagocytosis involves the Fcγ receptor, which mediates phagocytosis of IgG-opsonized (i.e., IgG - coated) bacteria [21]. No evidence exists however that fibronectin directly binds to either β2 integrin or the Fcγ receptor. A report suggested that β3 integrin is able to induce phagocytosis in insect cells [22]. Apoptotic cells marked with the soluble glycoprotein called milk fat globule-EGF factor 8 (MFG-E8) were phagocytosed by macrophages through αvβ3 [23]. Furthermore, an interaction between β1 and β3, both of which bind to fibronectin, has been documented, whereby αvβ3 is required to allow α5β1-mediated phagocytosis [24]. Thus, while fibronectin supports phagocytosis and can bind to integrins involved in phagocytosis, it is not known whether its isoforms containing EDA and EDB play any role in phagocytosis and if they do, which receptors are involved.

In this paper, we show that EDB-containing fibronectin is elevated in the cerebrospinal fluid of patients with bacterial meningitis. Indeed, phagocytosis is associated with increased production and release of EDB fibronectin, whereby this isoform by itself is able to enhance phagocytosis by up to 40 % compared to untreated cells. This effect is mediated through β3 integrin in cooperation with β2-mediated phagocytosis. Furthermore, deletion of β1 does not diminish phagocytosis as suggested by the literature [24]. Instead, it increases β2 and β3 expression on the surface of polymorphonuclear cells and hence increases phagocytosis. This paper thus shows a novel role for the fibronectin isoform containing EDB and offers new insights on the role of integrins in phagocytosis.

## Patients, materials, and methods

### Human samples

Cerebrospinal fluid from patients with meningitis or controls with headache who received lumbar puncture for diagnostic purposes was collected in the Neurology Department at the University of Heidelberg. Sample rests were examined for total fibronectin and the various isoforms after obtaining informed consent. The first cohort consisted of six patients with headache and six patients with bacterial meningitis. No further data are available. The second confirmatory cohort consisted of 14 patients with headache and 22 patients with bacterial meningitis with the following pathogens: five patients had proven Staphylococcus species, eight had *Streptococcus pneumoniae*, four had other Streptococcus species, one had *Listeria monocytogenes*, and four had *Neisseria meningitides*. Samples for in vitro evaluation of phagocytosis were collected at the Institute of Immunology of the University of Heidelberg after obtaining informed consent.

### Mice

Mice possessing an Mx or albumin promoter driving Cre recombinase expression were crossed with mice carrying *loxP*-flanked (floxed) fibronectin [25] or floxed β1 integrin genes [26]. Mx was induced at 3 weeks with three injections of polyinosinic–polycytidylic acid (250 μg/mouse) (Amersham) [25]. Plasma fibronectin was determined by ELISA, and β1 integrin deletion was determined by staining for β1 integrin and flow cytometry of total blood from tail-vein blood [27]. β3^−/−^ mice were obtained from mating heterozygote β3^+/−^ mice [28]. Their genotype was tested by PCR, and knockout confirmed by flow cytometry of peripheral blood. Terminal bleeding was performed at the age of 6–8 weeks. Animal studies were approved by the regulatory authorities (Regierungspräsidium Karlsruhe of the State of Baden-Württemberg).

### Cell isolation and phagocytosis induction

Blood was drawn from human subjects or mice in heparin-containing vials. Red blood cells were lysed by using 25 ml for 1 ml blood of hypotonic NaCl 0.2 %, mixing briefly (10 s), stopping with another 25 ml 1.6 % NaCl (to get an isotonic solution and prevent lysis of other cells), and centrifugation for 5 min at 490×*g*. Lysis was repeated thrice. An average of 1 ml blood was obtained per mouse and a total of 10 ml from healthy human subjects. One millimeter provides around 2 × 10^6^ cells. The pellet was resuspended in HBSS (Gibco). FITC-conjugated *S. aureus* (Wood-strain without protein A, Bioparticles, concentration 3.5 × 10^9^/ml, Life technologies) were opsonized by suspending with 10 % mouse or human heparin-plasma in HBSS and gently mixing for 20 min at 37 °C. Alternatively, IgG was used for the same duration to opsonize the bacteria at 2 mg/ml in HBSS (gamma-globulin, human, Sigma-Aldrich). Bacteria were centrifuged, washed with HBSS once, added to the cells at a ratio of 10:1, gently mixed, and incubated for 45 min for mice cells and 10 min for human cells at 4 or 37 °C. A control sample was not exposed to bacteria and left at 4 °C. Samples were quenched by adding crystal violet (2 g/l in 0.15 M NaCl) or trypan blue (Gibco), which allows the evaluation by flow cytometry (LSR2, BD) of the percentage of cells that phagocytosed the labeled bacteria. The supernatant was stored at −80 °C until analysis. Cells were either treated with TriFAST (Peqlab) for future RNA analysis or with Triton X-100 lysis buffer (20 mM, 150 mM NaCl, 10 % glycerol, 0.5 % Triton X-100, 2 mM EDTA, 10 mM NaF, 1 mM PMSF, and 1 mM Na_3_VO_4_) for future protein analyses of the cells.

Phagotest (Glycotope Biotechnologies) was performed as suggested by the manufacturer with one modification. Briefly, blood was obtained in heparin vials, and 100 μl were vortexed and kept on ice for 10 min. Bacteria (20 μl *Escherichia coli* from the kit or 2 × 10^7^*S. aureus* not opsonized) were added. The mixture was left 10 min at 37 °C, while the control was left on ice. The samples were then placed on ice for another 10 min, quenched with 100-μl quenching solution, vortexed, and washed twice. Lysis buffer was added to the pellet, samples vortexed and left at RT for 20 min. Centrifugation at 250x*g* at 4 °C was followed by two more wash steps. Lastly, 200-μl DNA staining solution was added to the pellet, sample vortexed and put on ice and measured by flow cytometry after 10 min.

All fibronectin isoforms were added at a final concentration of 20 ng/ml. Phorbol 12-myristate 13-acetate (PMA) was used at 16 ng/ml (SIGMA) to induce degranulation, the inhibitory monoclonal antibody directed against β2 (CD18) (Beckman Coulter/Immunotech, IM1567, clone 7E4) at a concentration of 4 μg/ml [29], the inhibitory antibody directed against αvβ3 at 10 μg/ml (clone LM609, Merck Millipore), and as a control for both MOPC 21 (Sigma) at the appropriate concentrations.

### Flow cytometry

Cells were collected and red blood cells lysed as described for the phagocytosis. The pellet was then resuspended in 100 μl FACS buffer (2.5 % FCS in D-PBS) and stained for 30 min at 4 °C with species-specific antibodies at a dilution of 1:100. Murine antibodies used were CD18 (Integrin-ß2): PE rat anti mouse, clone: M18/2 (Biolegend); CD29 (Integrin-ß1): PE armenian hamster anti mouse, clone: HMß1-1 (Biolegend); and CD61 (Integrin-ß3): PE armenian hamster anti mouse, clone: HMß3.1 (AbD Serotec). Human samples were stained with the following antibodies: CD29 (Integrin-ß1): Alexa Fluor 700 mouse anti human, clone: TS2/16 (Biolegend) and CD61 (Integrin-ß3): Alexa Fluor 647 mouse anti human, clone: VI-PL2 (Biolegend). Cells were then centrifuged, washed once, and then resuspended in 100 μl buffer for analysis at the LSR2 (BD) with the appropriate channels. For evaluation of actin polymerization, phalloidin staining was performed as follows: after red blood cell lysis, PMNs were incubated with opsonized bacteria in the presence of the different substances for 10 min at 37 °C, followed by fixation for 10 min with 4 % PFA, washing and staining with phalloidin Alexa-647 (Invitrogen #A22287) in PBS 1:40 for 30 min at RT. Cells were washed and mean fluorescence intensity measured.

### Production of EDB, control fibronectin, and EDA

We introduced a construct containing the total fibronectin complementary DNA (cDNA) as well as the EDB domain, but not the EDA domain in a cancer cell line (MDA-MB-231) in which fibronectin was previously deleted using 5′ UTR-specific short hairpin RNA (*sh*RNA) to delete endogenous fibronectin such that this line can only produce fibronectin containing the EDB domain as defined by the construct (FN^EDB+^ also called EDB). As a control, we introduced the total fibronectin cDNA that lacks EDA and EDB in the same cancer cell line (FN^EDB-^ also called plasma fibronectin or pFN). A clone containing only the EDA, but not the EDB domain, was also produced and introduced into the cell line (EDA). A single clone was selected, conditioned media were collected, fibronectin purified by affinity chromatography, identity confirmed by protein gel electrophoresis, and ELISA and the amount of fibronectin quantified.

### Staining protocols and immunohistochemistry

To determine whether fibronectin is found in specific granules, PMNs isolated as described were fixed with 4 % PFA, permeabilized with Triton X 100 0.2 % for 2 min, stained for fibronectin with sheep anti-fibronectin antibody (1:100, # Gentaur OBT0683), and then counterstained using donkey anti-sheep antibody labeled with Alexa-555 at 1:500 (Thermo Fischer A-21436). Lactoferrin was used to stain granules (1:100 of mouse IgG1 anti-human from Acris, 1 mg/ml, BM568) followed by a secondary goat anti-mouse antibody labeled with Cy2 from Dianova (115225166 at 1:500) each for an hour. ProLong Diamond anti-fade mounting medium (Molecular probes) was used, slides dried and evaluated by microscopy. The experiments on the relationship of EDB fibronectin, β3 integrin, and bacteria were performed as follows: PMNs were exposed to IgG-opsonized bacteria for 30 s at a ratio of 10:1, fixed and stained for EDB fibronectin using the BC1 antibody (mouse anti human, Antisoma #FN7b89) at 1:100 followed by a secondary goat anti-mouse antibody labeled with Cy3 (Dianova 115116062, 1:500), β3 integrin (CD61) was stained using a rabbit polyclonal anti-CD61 antibody at 1:50 (Millipore AB1932) and goat anti-rabbit Alexa-647 antibody (1:500, Abcam ab150079). Primary antibodies were added together for 60 min at 4 °C, and secondary antibodies for another 60 min. Nuclei were stained using DAPI. Cells were centrifuged, washed, and a drop was added for inverse fluorescence microscopy (ECLIPSE Ti, Nikon). Fibronectin staining is presented in red in Fig. 5a. β3 integrin labeled with Alexa-647 was pseudo-colored green to allow visualization of colocalization of fibronectin and β3 in yellow. The bacteria are FITC-labeled, but were pseudo-colored white.

### RNA analysis

RNA was isolated using TriFAST (Peqlab), and reverse transcribed using iScript-Select (BioRad). Quantitative PCR (qPCR) was performed using SensiMix™ Capillary Kit (Bioline), and results were normalized to murine or human HPRT. The primers used were those suggested by Roche universal probe library with modifications as follows: murine EDB (Probe 31), 5′: cccctatctctgataccgttgt, 3′: gaatcacagtagttgcggca; murine EDA (Probe 77), 5′: ttgcacgatgatatggagag, 3′:aggcataaagccactgttcc; murine fibronectin (Probe 66), 5′: tttgctcctgcacgtgttt, 3′:ctgtgtatactggttgtaggtgtgg; human EDA (Probe 32), 5′: ttgcacgatgatatggagag, 3′: aattcattcagtagggcataaagc; human EDB (Probe 31), 5′: tttccctctattttccttttgcc, 3′: ctgccgcaactactgtgatg; human fibronectin (Probe 76), 5′: actgagactccgagtcagcc, 3′: ttccaacggcctacagaatt.

### Protein analysis

For mass-spectrometry, 100 μg proteins, i.e., 5 μl from the meningitis sample with the highest EDB and 20 μl from the control sample with the lowest EDB, were run on a 2-D gel. Five spots stained for EDB fibronectin (using BC1-clone) and present only in the meningitis sample were evaluated by mass-spectrometry with the question: can the sequence for EDB fibronectin be identified, which it did [30]. For western blot analysis, the following antibodies were used: murine anti-EDB (clone BC1, courtesy D. Neri), rabbit anti-fibronectin (Millipore), rabbit anti-EDA (clone FN3E2), rabbit GAPDH (Sigma), ERK, pERK, AKT, pAKT (Cell signaling), anti mouse-HRP (BioRad), and anti rabbit-HRP (Dianova). All antibodies were diluted 1:1000 except for GAPDH, which was used at a dilution of 1:10 000. Samples were loaded after adjusting to protein content measured by BCA (Pierce).

### ELISA

Fibronectin was quantified in mouse plasma, cell lysates, and conditioned media by ELISA as reported [27, 31] and corrected to protein content measured by BCA (Pierce) when appropriate. Briefly, plates were coated with the primary antibody (0.12 μg/ml) (F3648, Sigma). For mouse plasma, lysates, and conditioned media, the standard used was for mice murine plasma fibronectin (#IMFBN, Dunn) or for human samples human plasma fibronectin isolated as described [2]. As a secondary antibody, anti-fibronectin-HRP-conjugated antibody (P0246, DAKO) was used. For EDA ELISA, the plates were coated with a primary antibody at 1.195 mg/ml (FN-3E2, Sigma). For EDB, the primary antibody was kindly provided by Dr. D. Neri (Swiss Federal Institute of Technology (ETH)) and applied to the plates at a concentration of 2.5 μg/ml, L19SIP) [7]. The secondary antibody used was the same as for total fibronectin. The standard was established in our lab after purifying the isoforms EDA and EDB using antibody columns and quantifying fibronectin content.

### Statistical analyses

Analyses were performed using SPSS (V20). ANOVA and repeated-measures ANOVA tests were used as appropriate. If global probability values were smaller than 5 %, subsequent comparisons between selected group pairs were then performed using Student’s *t*, Mann–Whitney, or Wilcoxon paired tests as appropriate. Pearson correlations were estimated to evaluate the relationship between the two variables. Results are expressed as mean ± standard error of the mean (M ± SEM).

## Results

### Bacterial meningitis is associated with increased EDB-containing fibronectin

Fibronectin enhances phagocytosis of bacteria by immune cells [16, 17] and is increased in patients with bacterial meningitis [32, 33]. We therefore asked whether we could confirm these changes and whether the isoforms containing EDA or EDB are also increased in bacterial meningitis.

**Fig. 1:**
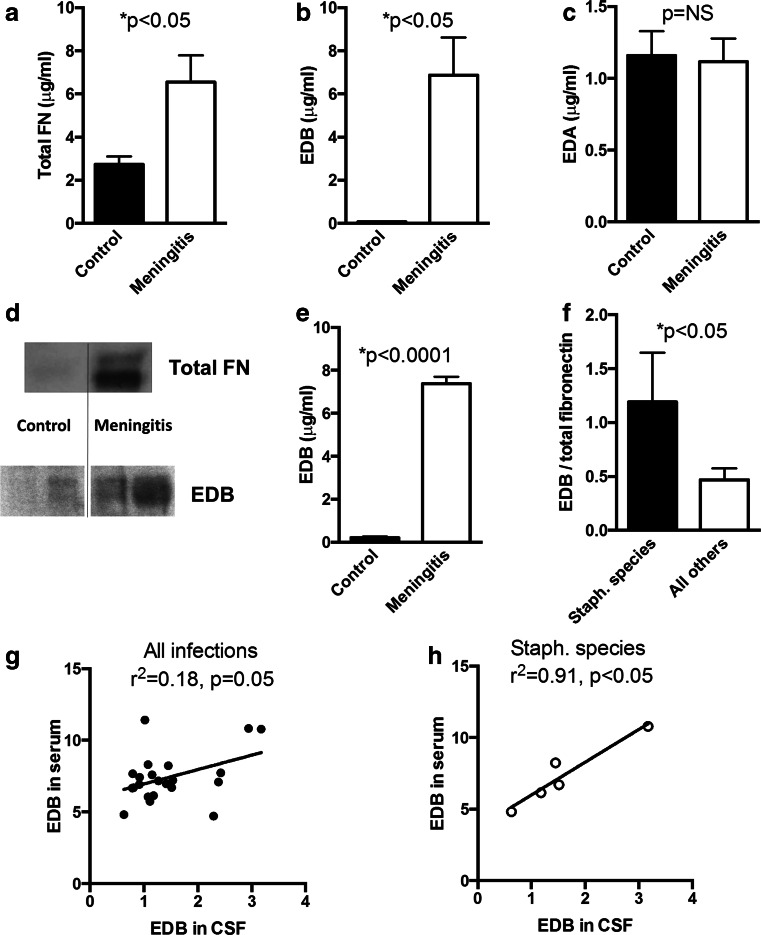
Meningitis results in elevation of EDB-containing fibronectin (EDB) in the cerebrospinal fluid (CSF). **a** Total fibronectin (total FN) is elevated in CSF of patients with bacterial meningitis 2.4-fold compared to healthy controls (*n* = 6 + 6). **b** EDB-containing fibronectin (EDB) is elevated in CSF of patients with bacterial meningitis 87-fold compared to healthy controls. **c** EDA-containing fibronectin (EDA) does not differ between patients with bacterial meningitis and healthy controls. CSF obtained by lumbar puncture was tested by ELISA using specific antibodies. Results represent the mean of six patients per group. **d** Western blot analysis confirms an increase in total fibronectin (FN) and EDB fibronectin in CSF from patients with bacterial meningitis. Protein content was measured by BCA, and the same amount of CSF was added in both wells in the top two lanes, while the same amount of total fibronectin as determined by ELISA was added in the bottom four wells (two replicates for controls and tow for meningitis are shown). FN results in two bands because it consists of a dimer. **e** EDB fibronectin was increased in a larger cohort of 14 controls (CT) and 22 patients with meningitis. **f** EDB/total fibronectin (%) is higher in patients with staphylococcus species infections (5 vs. 17). **g** EDB fibronectin in CSF shows a poor relationship with EDB fibronectin in serum in the whole cohort (22 patients). **h** In the patients with staphylococcus infections (*n* = 5) both correlate well

We quantitated fibronectin and its isoforms in the cerebrospinal fluid (CSF) and found a 2.4-fold increase in the amount of total fibronectin in the CSF of six patients with bacterial meningitis compared to six controls (CT) with non-infectious headaches (Fig. 1a). EDB-containing fibronectin was 80-fold increased in patients with bacterial meningitis, while EDA-containing fibronectin did not differ (Fig. 1b–c). The presence of the EDB domain detected by ELISA was further confirmed by Western blotting (Fig. 1d) as well as 2-D gel electrophoresis followed by mass spectrometry for the spots detected in the meningitis samples but not in the control samples.

We then confirmed these findings in a larger group (14 controls with headache and 22 bacterial meningitis patients) (Fig. 1e). In addition, EDB-containing fibronectin in the CSF was 5.9-fold higher compared to EDB-containing fibronectin in peripheral blood in patients with meningitis while the ratio was much lower in healthy subjects (CT: 0.2+/−0.02 vs. meningitis: 5.9+/−0.5 fold, *n* = 14 + 22, *p* < 0.0001). We next asked whether the increase in EDB-containing fibronectin in the CSF was related to the severity of the disease or to the involved pathogen. The modified RANKIN score at admission (higher values reflect worse disease) did not show a relevant correlation with the ratio of EDB/total fibronectin in the whole group of patients with meningitis (*r*^2^ = 0.22, *p* = 0.05, *n* = 22) [34]. In patients with staphylococcal infections, however, almost all of the fibronectin detected in the CSF contained the EDB domain in contrast to other patients in which only about half of fibronectin contained the EDB domain (Fig. 1f). In addition, in this cohort, the concentration of EDB-containing fibronectin in the CSF was closely related to its level in the serum (*r*^2^ = 0.91, *p* < 0.05, *n* = 5), which was not the case in the whole cohort (Fig. 1g–h).

Thus, in bacterial meningitis, EDB-containing fibronectin is increased in the cerebrospinal fluid, and this increase is related to the pathogen, whereby staphylococcal infections result in more EDB-containing fibronectin in the CSF.

### Phagocytosis of bacteria is associated with increased EDB production and release

**Fig. 2:**
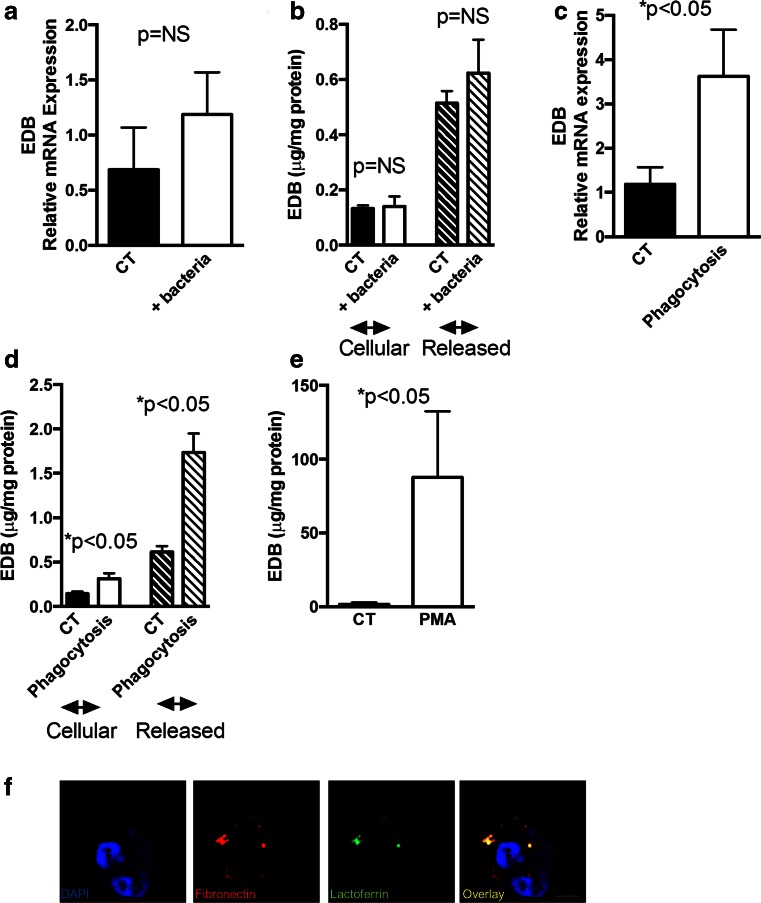
Phagocytosis is associated with the production and the release of EDB fibronectin. **a** The presence of bacteria does not significantly affect the production of EDB at the mRNA level. Cells were obtained from six healthy subjects and either left untreated or subjected to the addition of bacteria and left at 4 °C for 45 min. EDB was measured by qPCR corrected to the housekeeping gene HPRT. **b** EDB-containing fibronectin in the cell lysates and in the media was measured by ELISA and corrected to the protein content of the cells measured by the BCA method. N as in A. **c** Phagocytosis results in an increase in EDB mRNA. Cells were treated with *Staphylococcus aureus* and left at 37 °C for 10 min and compared to cells treated with *S. aureus*, but left at 4 °C. N as in A. **d** Phagocytosis is associated with an increase in EDB-containing fibronectin in the cell lysate or released in the media. **e** PMA, which induces degranulation, results in increased EDB fibronectin in the media as measured by ELISA. *N* = 3. **f** Fibronectin partially colocalizes with lactoferrin found in specific granules. PMNs were isolated, fixed, permeabilzed, and stained for fibronectin in *red*, lactoferrin in *green*, and DAPI in *blue* to visualize the nuclei. Bars represent 2.5 μm

Bacterial meningitis is usually associated with increased white blood cell numbers in the cerebrospinal fluid (CSF) [35]. In particular, granulocytes (or polymorphonuclear leukocytes: PMN) increase both in absolute and relative numbers [35] and produce EDB-mRNA [36]. Therefore, these cells represent a possible source for EDB fibronectin protein in the CSF. To evaluate for this possibility, opsonized *S. aureus* particles were added to human PMNs. Exposure of PMNs to bacteria at 4 °C did not significantly affect EDB mRNA expression, the amount of EDB fibronectin detected in the cell lysates or released to the media (Fig. 2a–b). In contrast, incubation of PMNs with opsonized bacteria at 37 °C increased EDB mRNA expression, as well as both the amount of EDB-containing fibronectin protein found in the cell lysate and released into the media compared to CT cells (Fig. 2c–d) suggesting that active phagocytosis and not the mere exposure to bacteria increases EDB fibronectin production and release. Treatment of PMNs with a non-toxic dose of PMA (phorbol 12-myristate 13-acetate) for 10 min resulted in release of EDB fibronectin. Since PMA induces degranulation, this finding is compatible with release of EDB fibronectin from the granules (Fig. 2e). Furthermore, co-staining of lactoferrin (expressed in specific granules) [37, 38] and fibronectin confirmed partial colocalization (Fig. 2f).

Thus, phagocytosis is associated with increased EDB fibronectin production and release.

### Fibronectin originating from the immune cells contributes to phagocytosis

**Fig. 3:**
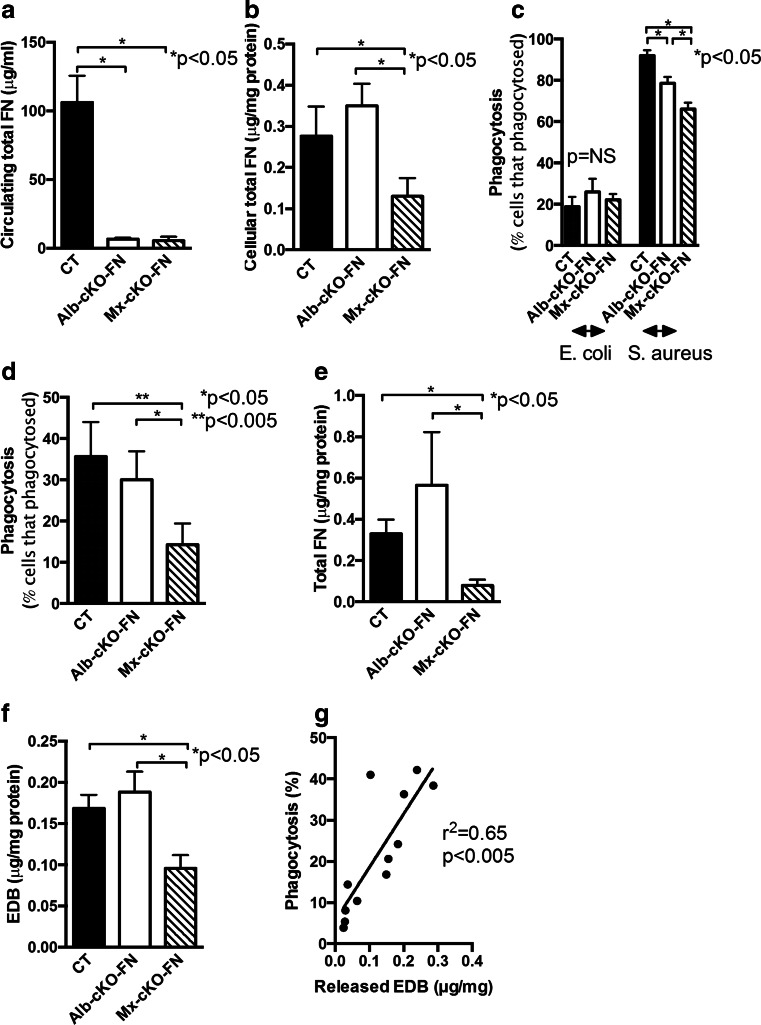
The absence of immune cell fibronectin diminishes phagocytosis. **a** Circulating total fibronectin (FN) is diminished when fibronectin is deleted in the hepatocytes (Alb-cKO-FN) to a similar degree as the decrease when fibronectin is deleted in the hepatocytes and in the immune cells (Mx-cKO-FN). Blood obtained from the tail vein was examined by ELISA. *N* = 7–10 mice per group. **b** Cell lysates show a decrease in total fibronectin and in EDB fibronectin in Mx-cKO-FN mice, but not in Alb-cKO-FN mice as determined by ELISA of the cell lysates and correcting to total protein measured by BCA. *N* = 5/group. **c** In a phagocytosis test (Phagotest) where bacteria are added to total blood and phagocytosis is determined after 10 min, deletion of circulating fibronectin diminished phagocytosis of *S. aureus* (Alb-cKO-FN), but this decrease was more pronounced when both circulating and immune cell fibronectin were deleted (Mx-cKO-FN). Phagocytosis of *E. coli* was not affected by fibronectin availability. **d** In a phagocytosis test in which the cells are separated from the serum and the bacteria opsonized with control serum, phagocytosis with cells from Alb-cKO-FN is similar to CT. Deletion of fibronectin in the immune cells in Mx-cKO-FN results in a significant decrease in phagocytosis. *N* = 5 pools of blood from 3–5 mice/pooled group. **e**–**f** The decrease in phagocytosis is associated with a decrease in total (**e**) and EDB fibronectin (**f**) released in the media and corrected to protein. N as in D. **g** The levels of EDB fibronectin released in the media correlates with the degree of phagocytosis in CT mice. *N* = 12 pools of blood from 2–3 mice/pooled group

We next evaluated whether EDB fibronectin itself modulates phagocytosis. To test this, we deleted fibronectin in immune cells using the cre/lox*P* system, which allows for deletion of fibronectin in the desired cell types. Since fibronectin is sticky and attaches to most surfaces including immune cell surfaces, simultaneous deletion of fibronectin in the circulation and in the immune cells would be best for our purposes but requires a control in which only circulating fibronectin is deleted. Deletion of circulating fibronectin only can be achieved by using the albumin promoter attached to Cre recombinase in mice that carry floxed fibronectin genes on both alleles (Alb-cre_FN^fl/fl^). The albumin promoter becomes activated in hepatocytes only, and these cells contribute almost all of circulating fibronectin. The production of Cre recombinase in hepatocytes thus results in deletion of fibronectin in the circulation (Albumin-driven conditional knockout: Alb-cKO-FN). To delete fibronectin in both the circulation as well as in the immune cells, the Mx promoter attached to Cre was used (Mx-cKO-FN), and Mx activated 3 weeks prior to the experiments (Fig. 3a–b) [26]. These two models thus allow the differentiation between the effect of deletion of fibronectin in the circulation (Alb-cKO-FN) or deletion in both the circulation and the immune cells (Mx-cKO-FN).

We first evaluated the phagocytic capability of the immune cells in the presence of the mouse own plasma. Therefore, no fibronectin-mediated enhancement of phagocytosis takes place in cKO mice. This makes phagocytosis dependent on the presence of other opsonins in the plasma. Phagocytosis of *S. aureus* was significantly diminished in the absence of circulating fibronectin in Alb-cKO-FN mice. Deletion of fibronectin in the immune cells in addition to the circulation was associated with a further decrease in phagocytosis as shown in Fig. 3c. Interestingly, fibronectin did not affect phagocytosis of *E. coli* measurably.

In order to establish that the difference in phagocytosis between Alb-cKO-FN and Mx-cKO-FN mice is dependent on immune cell fibronectin, we evaluated phagocytosis in isolated PMNs from the various genotypes (after separation from the plasma). Added bacteria were opsonized with control plasma from wild-type mice. Using this method, we detected a significant difference in the degree of phagocytosis between Alb-cKO-FN and Mx-cKO-FN (Fig. 3d), associated with a difference in the release of total and EDB-containing fibronectin (Fig. 3e–f). Indeed, we confirmed a positive correlation between the efficiency of phagocytosis and release of EDB fibronectin in vitro (*r*^2^ = 0.65, *p* < 0.005), which suggests that phagocytosis is increased whenever EDB fibronectin release is higher (Fig. 3g).

Thus, fibronectin originating from the immune cells affects the phagocytic function of these cells.

### T-cells are not involved in affecting phagocytosis in the absence of fibronectin

**Fig. 4:**
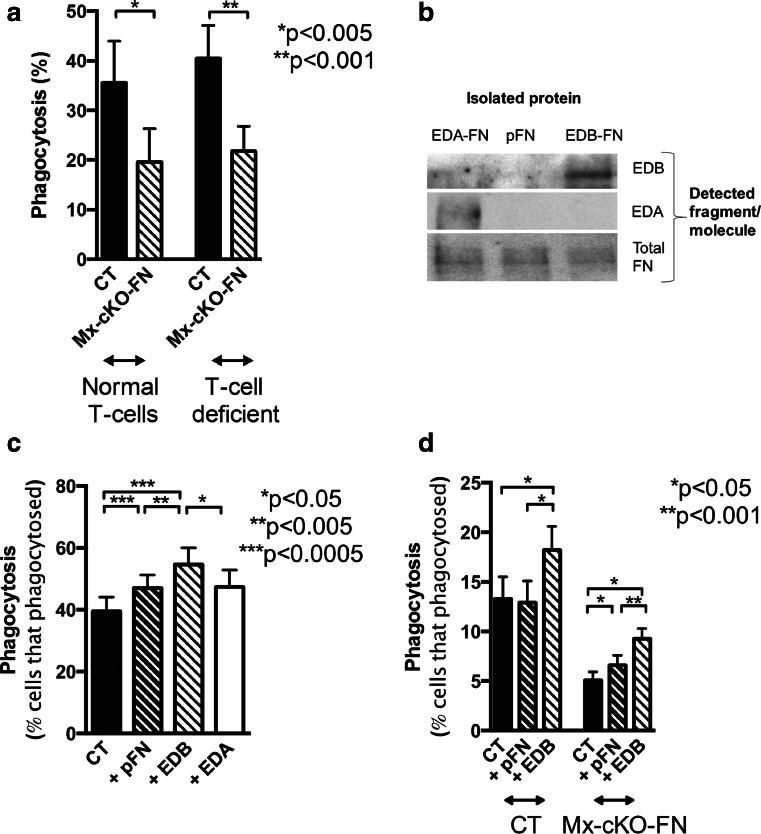
T-cells do not affect phagocytosis in the absence of fibronectin. **a** Independent of whether immune cells produced fibronectin or not, the absence of T-cells did not measurably affect phagocytosis. *N* = 2–5 mice per measurement and six measurements/group. EDB-containing fibronectin enhances phagocytosis. **b** The presence of the EDB domain, but not the EDA domain was confirmed in isolated EDB fibronectin (FN) by western blotting. Total fibronectin was detected in the three isolated isoforms. Same amount of fibronectin was added in the wells of each gel and blotted with either an antibody directed against EDA, against EDB or against total fibronectin as shown on the right. In the gel for total fibronectin, the amount of fibronectin added was 4-fold less than for the isoforms. **c** Adding control fibronectin (plasma fibronectin: pFN) slightly increased phagocytosis in human PMNs, but adding EDB fibronectin prior to adding *S. aureus* enhances phagocytosis. EDA fibronectin failed to enhance phagocytosis. Cells were isolated from four healthy subjects and treated with plasma-opsonized *S. aureus*. Prior to adding the bacteria to the cells, pFN or EDB fibronectin was added at a concentration of 200 ng/ml, which is 5-fold the concentration of EDB fibronectin in CSF. Phagocytosis measured after incubating the cells for 10 min at 37 °C and compared to cells treated similarly but left at 4 °C instead. *N* = 13 replicates except for EDA: five replicates. **d** Cells from control (CT) mice are not affected by adding pFN, but EDB fibronectin enhances phagocytosis. Mx-cKO-FN mice show enhanced phagocytosis already by addition of pFN but EDB fibronectin effect is more pronounced. Cells were similarly isolated and treated for 45 min. *N* = 7 pools of blood from four to six mice each/pooled group

T-cells produce EDB mRNA [39]. To evaluate whether T-cells affect phagocytosis, we used *Foxn1*^*mut*^ mice (nu/nu mice). Homozygote mice are athymic and hence unable to develop thymus-derived T-lymphocytes. Because the absence of fibronectin in immune cells suppressed phagocytosis, we mated the mice such that we were able to test the effect of loss of T-lymphocytes in the presence and absence of fibronectin production by the immune cells comparing controls (CT nu/nu) with Mx-cKO-FN nu/nu mice. As shown in Fig. 4a, phagocytosis was similar in the presence or absence of T-lymphocytes in mice with normal fibronectin levels. Furthermore, deletion of fibronectin using the Mx-promoter resulted in a similar decrease in phagocytosis independent of whether mature T-cells were present or not.

Taken together, the loss of fibronectin in immune cells results in decreased phagocytosis independent of the presence or absence of thymus-schooled T-lymphocytes.

### EDB fibronectin enhances phagocytosis both in the presence and absence of plasma fibronectin

In order to determine whether a causal relationship between EDB fibronectin and enhanced phagocytosis exists, we sought to isolate pure fibronectin that either contains EDB or lacks it. To achieve this, we first deleted endogenous fibronectin in a cancer cell line using 5′ UTR-specific *sh*RNA. We then introduced a construct containing total fibronectin cDNA including the EDB domain, but not the EDA domain. This line can thus only produce fibronectin containing the EDB domain as defined by the construct (FN^EDB+^ was called EDB). As a control, we introduced the total fibronectin cDNA that lacks EDA and EDB in the same cell line (FN^EDB-^: pFN, because it resembles the circulating plasma fibronectin), as well as a construct that contains the EDA but not the EDB domain (which we called EDA in the figure). Single clones were selected, conditioned media were collected, fibronectin purified by affinity chromatography, the amount of fibronectin quantified, and identity confirmed by ELISA and protein gel electrophoresis (Fig. 4b).

Opsonized bacteria were added to human PMNs at the same time as fibronectin lacking EDB or EDA (pFN) or containing EDB (EDB) or containing EDA (EDA). EDB addition enhanced phagocytosis (Fig. 4c). The same effect was seen in murine cells. The difference, however, was more pronounced in Mx-cKO cells, which already had some response to pFN, the control molecule. Presumably, this is due to the lack of fibronectin production by these cells and the ability of plasma fibronectin (pFN) to boost phagocytosis (Fig. 4d).

In summary, adding EDB-containing fibronectin augmented phagocytosis more than control fibronectin lacking EDB (pFN).

### β3 integrin interaction with EDB fibronectin contributes to enhancement of phagocytosis

**Fig. 5:**
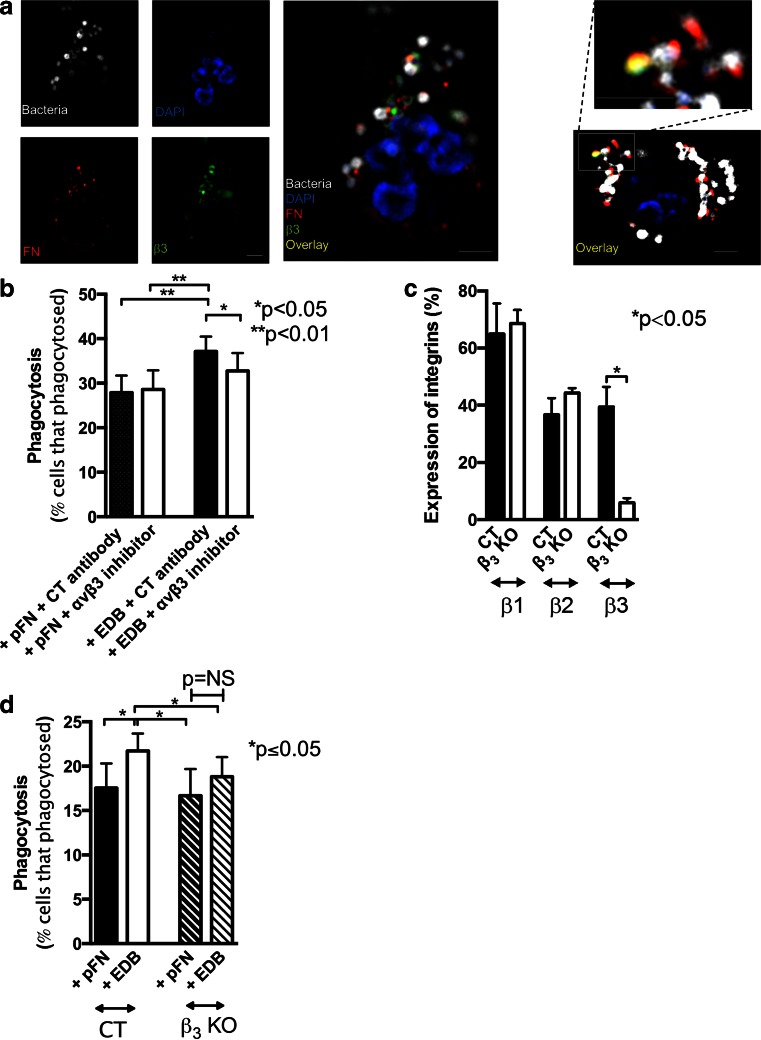
EDB fibronectin enhances phagocytosis through activating β3 integrin. **a** EDB fibronectin (FN) in *red*, β3 integrin in *green*, and bacteria in *white* are found in the proximity of each other. DAPI (*blue*) was used as a nuclear stain. PMNs were exposed to opsonized bacteria for 30 s, followed by fixation and staining. On the left, details of one cell are shown. On the right, a second example with a higher magnification of a clump of bacteria with fibronectin and β3 integrin co-staining is shown. *N* = 3 experiments. Bars represent 2.5 μm. **b** Using an αvβ3 inhibitory antibody results in diminished EDB-mediated phagocytosis (concentration used, 10 μg/ml). *N* = 8. **c** In β3 knockout mice, the percentage of PMNs expressing β1 or β2 integrin is similar, but β3 is deleted. **d** In the absence of β3, phagocytosis is no longer enhanced in the presence of EDB fibronectin (*n* = 5 pairs)

No receptor was yet identified for EDB fibronectin in cells. However, binding between an EDB-containing fibronectin fragment and αvβ3 was reported in electron microscopy studies using integrin molecules and EDB fragments that also contain the RGD sequence [40]. This raises the possibility that integrin αvβ3 mediates EDB fibronectin effects on phagocytosis. In line with this notion, added bacteria were localized in the proximity of both EDB fibronectin and β3 integrin (Fig. 5a). Using an inhibitory antibody specific for αvβ3 integrin did not affect pFN-mediated phagocytosis, but diminished the enhancement of phagocytosis by EDB fibronectin (Fig. 5c). Finally, we used PMNs isolated from β3 knockout mice (β3^−/−^: β3 KO) and confirmed deletion of β3 integrin, but no change in either β2 or β1 integrins on the cell surface (Fig. 5d). In these cells, EDB failed to enhance phagocytosis significantly in six experiments (Fig. 5e).

Based on these data, we conclude that the interaction of EDB-containing fibronectin with αvβ3 integrin contributes to phagocytosis.

#### The role of Fcγ receptors

**Fig. 6:**
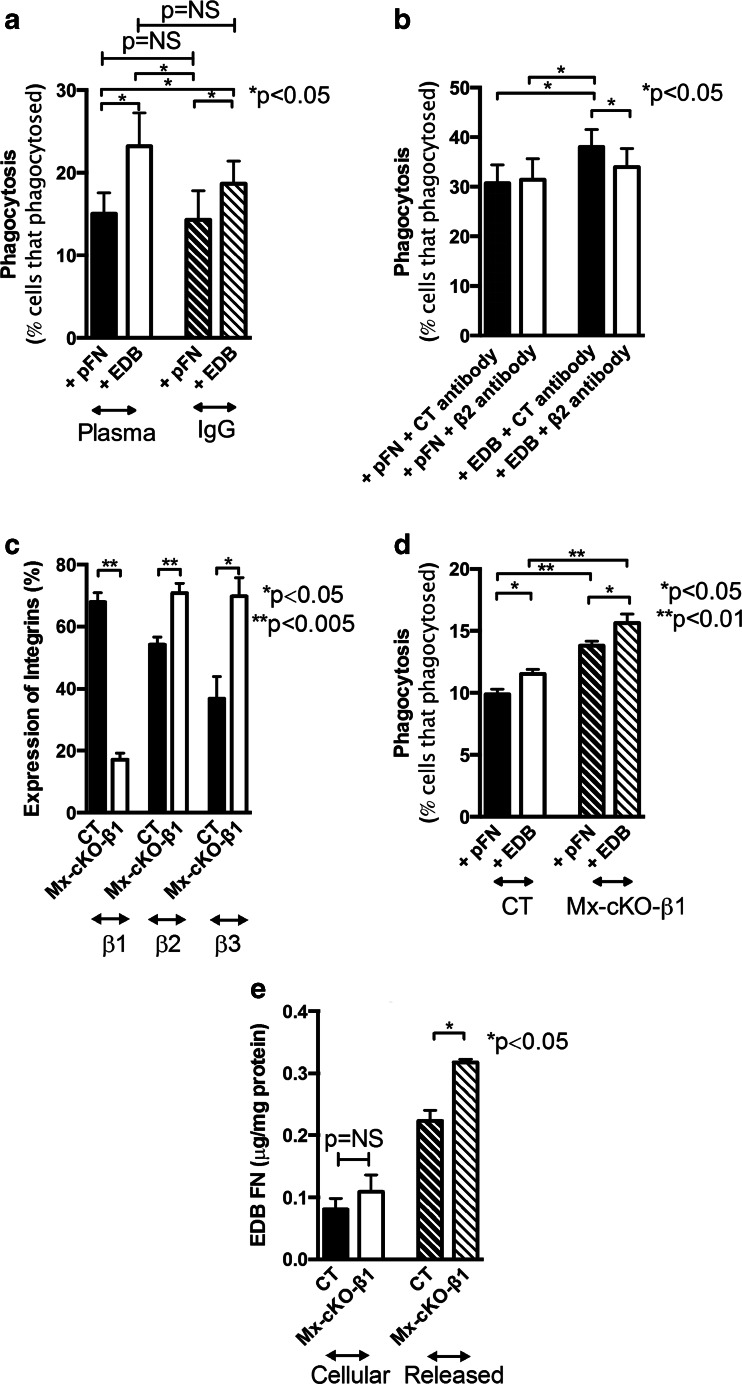
Mechanistic studies on the effect of EDB fibronectin and the interaction with other receptors. **a** Role of Fcγ receptor: Human PMNs were exposed to bacteria opsonized with plasma or with IgG. EDB fibronectin resulted in similar enhancement of phagocytosis. *N* = 5. **b** Role of α_M_β2: Inhibiting β2 (using an inhibitory antibody) decreases phagocytosis despite the presence of EDB fibronectin. Human PMNs were subjected to phagocytosing plasma-opsonized *S. aureus* at 37 °C for 10 min. Prior to adding the bacteria, cells were treated with the antibodies (4 μg/ml), *N* = 7. **c** Deletion of β1 integrin is successful in Mx-cKO-β1 and is associated with an increase in β2 and β3 expression. Expression was determined by flow cytometry. *N* = 4 pools of blood from two to three mice/pooled group. **d** Deletion of β1 integrin results in enhanced phagocytosis that is further increased by EDB fibronectin administration. *N* = 4 pools of blood from two to three mice/pooled group. **e** EDB fibronectin release in the media is enhanced in Mx-cKO-β1 cells during phagocytosis. N as in D

We then investigated whether an interaction between EDB fibronectin and the Fcγ receptor could be documented. We therefore opsonized bacteria with IgG, which results in activation of the Fcγ receptor or plasma, which contains a variety of other opsonins in addition to IgG. Phagocytosis at baseline did not differ using the two opsonization methods (Fig. 6a). Furthermore, EDB fibronectin addition enhanced phagocytosis of bacteria to a larger degree than pFN alone, irrespective of whether bacteria were opsonized with IgG or plasma, suggesting that phagocytosis through the Fcγ receptor did not interfere with enhanced phagocytosis by EDB fibronectin (Fig. 6a).

#### The role of β2-containing integrins

Since α_M_β2 integrins are also involved in phagocytosis [18], we inhibited β2 and found diminished phagocytosis in EDB-treated cells, despite using a dose of the β2 inhibitor that did not affect phagocytosis in the presence of pFN (Fig. 6b). Thus, phagocytosis enhancement by EDB involves β2 integrin.

#### The role of β1-containing integrins

EDB fibronectin contains the RGD-binding site of fibronectin to integrins. It therefore can bind to α5β1, the classical fibronectin receptor. EDA fibronectin enhances binding to α5β1 integrin, but no experimental data on EDB fibronectin affecting β1 integrin have been reported. To test this, we deleted β1 integrin in immune cells using Mx-cre, as for deletion of fibronectin, in mice homozygous for floxed β1 integrin (Mx-cKO-β1) (Fig. 6c) [26]. Deletion of β1 integrin was associated with an increase in both β2 and β3 expressions (Fig. 6c). The absence of β1 integrin was associated with both enhanced release of EDB fibronectin and enhanced phagocytosis (Fig. 6d–e).

Taken together, these data show that EDB fibronectin interacts with αvβ3 to enhance phagocytosis, and α_M_β2 is involved in this effect. Therefore, inhibiting either one diminishes phagocytosis but does not suppress it back to baseline, while phagocytosis mediated by the Fcγ receptor could also be enhanced by EDB fibronectin.

### EDB fibronectin enhances phagocytosis by affecting actin polymerization and intracellular signaling

**Fig. 7:**
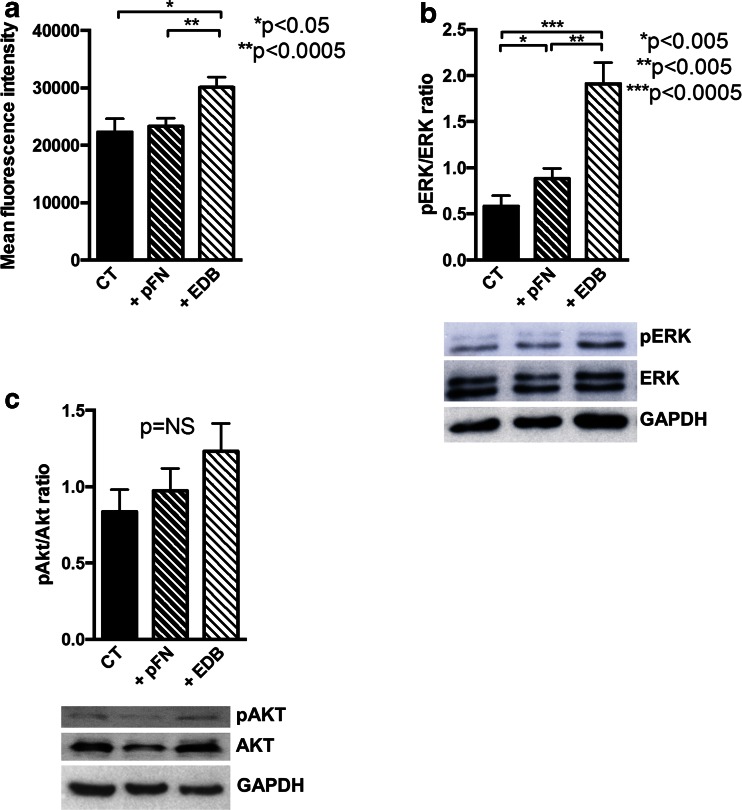
Studies on the effect of EDB fibronectin on actin polymerization and intracellular signaling. **a** Actin polymerization was evaluated by phalloidin staining in the presence of opsonized bacteria and pFN or EDB-containing fibronectin for 10 min and mean fluorescence intensity measured. *N* = 8. **b** pERK/ERK ratio is increased in PMNs treated with pFN and further enhanced with EDB fibronectin administration 10 min prior to cell lysis. *N* = 6. **c** An apparent trend to an increase in pAKT/AKT by Western blotting fails to reach significance in nine replicates/treatment. Human PMNs were prepared as for pERK/ERK ratio. GAPDH was run on a separate gel using the same amount of lysate for technical reasons

Phagocytosis requires actin microfilament formation and the activation of intracellular signaling cascades [12]. To investigate whether EDB fibronectin affected intracellular events differently from pFN, we evaluated phalloidin staining of polymerized F-actin and found that it was significantly enhanced by the presence of both bacteria and EDB (Fig. 7a). β2 (CD18) activation does not increase ERK phosphorylation [41]. Nevertheless, EDB increased ERK phosphorylation more so than pFN, which is in line with the increase in actin polymerization and suggests β3 activation (Fig. 7b) [42]. In contrast, AKT phosphorylation, which increases in response to α_M_β2 stimulation [43] failed to show statistically significant changes in nine biological replicates, despite what seemed to be a trend upwards (Fig. 7c).

Thus, EDB fibronectin enhances phagocytosis by activating β3 integrin and increasing ERK phosphorylation, without marked effects on AKT phosphorylation.

## Discussion

The principal findings of this study are (1) EDB-containing fibronectin is released in the cerebrospinal fluid of patients with bacterial meningitis, especially during infections with staphylococcal species. (2) Phagocytosis is associated with the production and release of EDB-containing fibronectin. (3) The presence of the EDB domain in fibronectin augments phagocytosis by acting on αvβ3 integrin. (4) This effect is independent of the presence of β1 integrin. (5) β2 seems to cooperate with β3 to enhance phagocytosis mediated by EDB fibronectin.

Fibronectin is elevated in the cerebrospinal fluid of patients with bacterial meningitis [44]. It was suggested that this aggravates inflammation and increases the damage [32]. Our data suggest that almost all the fibronectin released during infections with staphylococcal organisms contains the EDB domain and enhances the phagocytic response towards bacteria. The EDA isoform was diminished in children [45], but in our cohort this finding could not be confirmed. In addition, EDA fibronectin did not affect phagocytosis measurably in our model. It is conceivable, however, that the increase in the production and release of EDB fibronectin occurs at the expense of the production of EDA fibronectin in pediatric patients, but not in our adult subjects resulting in the discrepancy between the two cohorts.

We evaluated the role of fibronectin produced by the immune cells themselves. The Mx promoter deletes fibronectin and β1 integrin in hematopoietic cells. However, it also deletes fibronectin in the liver. For this reason, we used as the control the albumin promoter to delete fibronectin in hepatocytes and hence in the circulation [25, 26]. This allowed the clarification of the role of PMN fibronectin as opposed to the role of circulating plasma fibronectin, which, in our hands, is limited. The limited effect of circulating fibronectin may explain the contradictions reported in the literature on fibronectin involvement in phagocytosis [17, 46]. It should be noted that only a small percentage of total circulating fibronectin contains EDB [27], and therefore studies performed with fibronectin isolated from the plasma would have failed to point an effect [17, 46]. In addition, our data point to de novo synthesis of EDB-containing fibronectin during phagocytosis in PMNs (Fig. 2c–d), which is in line with published reports [36].

The presence of the EDB domain affects vasculogenesis, but some overlap exists with EDA in that function [4, 5]. No specific integrin that binds to the EDB domain was identified, except in a single in silico study where the interaction between a fragment containing EDB and αvβ3 integrin was reported [40]. This interaction however could take place at the RGD sequence, which is the known binding sequence of fibronectin to αvβ3 [1]. In this case, the presence of EDB would only change the characteristics of fibronectin binding to αvβ3, presumably in a manner similar to the role of EDA in enhancing binding of the RGD sequence to α5β1 integrin [47]. Our data demonstrate that αvβ3 integrin mediates EDB fibronectin effects in cells. EDB fibronectin enhances phagocytosis by augmenting β3-integrin-mediated signaling as evidenced by activating actin polymerization and increasing ERK phosphorylation. How EDB fibronectin affects phosphorylation of the cytoplasmic tail of β3 is not clear however [48]. Our data also confirm the role of this integrin reported in phagocytosis by insect cells in mammalian cells [22].

Integrins consist of α and β subunits that diffuse freely in the membrane and between an intracellular and a surface pool, making the integrin pool very dynamic. α and β subunits need to combine in heterodimers to affect intracellular signals [20]. Each β subunit can bind to a limited number of α counterparts, but some pairs are found more often than others. Binding of integrins to extracellular proteins then stabilizes the αβ-pair in focal adhesions [49]. The decrease in β1 on PMNs of Mx-cKO-β1 was associated with increased β3 expression on the cells (Fig. 6c), in line with the concept of a steady pool of integrin subunits available for expression in which a decrease in β1 allows αv to bind to other β subunits, in this case β3, as implied from the data of various groups [50–53]. Because EDB fibronectin acts via αvβ3, an increase in αvβ3 could contribute to the enhancement of phagocytosis by EDB fibronectin in β1 cKO or alternatively result in boosted EDB fibronectin release by yet undefined mechanisms (Fig. 6e). Interestingly, β2 expression on the cell surface was elevated in the β1 cKO too. It therefore cannot be ruled out that enhanced phagocytosis in these cells results from increased expression of either one (β2 or β3) or both subunits.

In our experiments, we used *S. aureus* strain Wood 46, which expresses relatively low amounts of fibronectin binding proteins. Nevertheless, it is able to bind fibronectin effectively as shown by others [54, 55] and confirmed by us (supplementary figure 1). Furthermore, this strain lacks protein A as does *E. coli* used in the experiment in Fig. 3c. Despite this common feature, EDB enhanced phagocytosis of *S. aureus*, but did not affect the phagocytosis of *E. coli*. This therefore suggests that protein A is not required for EDB effects.

Because of the importance of phagocytosis for survival, its control evolved such that much overlap exists and many possible pathways for activating phagocytosis can be simultaneously involved. Indeed, multiple receptors participate in phagocytosis, including Fc receptors, complement receptors, and pattern recognition receptors. For most bacteria species, opsonization with IgG and engagement of the Fc receptors is a prerequisite for efficient phagocytosis, while the other receptor-ligand interactions exert various modulatory roles [56]. Furthermore, a crosstalk sometimes takes place between the Fc receptor and integrins [57]. This work establishes the role of another modulator of phagocytosis of *S. aureus*, and clearly indicates that EDB fibronectin released during phagocytosis, especially of staphylococcus species enhances bacterial removal by mammalian immune cells through activating αvβ3 integrin. The experiments furthermore establish β3 integrin as the receptor mediating EDB fibronectin enhancement of phagocytosis. Our findings highlight that this mechanism acts in addition to other mechanisms of phagocytosis, but cannot be completely replaced by other phagocytic systems, at least in vitro. Lastly, this apparently important role played by αvβ3 integrin in phagocytosis needs to be taken into account in studies examining inhibitors of αvβ3 in cancer treatment.

## Electronic supplementary material

Below is the link to the electronic supplementary material.

ESM 1 (PDF 59 kb)
