# Supplementary material for: Identification and characterization of a unique role for EDB fibronectin in phagocytosis
Source: J Mol Med (Berl). 2015 Dec 5;94:567–81. doi: 10.1007/s00109-015-1373-0 (PMC4856727; doi:10.1007/s00109-015-1373-0)
Supplement: Supplementary file 1 — (PDF 59 kb) [file 109_2015_1373_MOESM1_ESM.pdf]

ELECTRONIC SUPPLEMENTARY MATERIAL

IDENTIFICATION AND CHARACTERIZATION OF A UNIQUE ROLE FOR EDB-FIBRONECTIN IN PHAGOCYTOSIS

Sabrina Kraft[1,2], Verena Klemis[1,2], Carla Sens[1,2], Thorsten Lenhard[3], Christian Jacobi[3,4], Yvonne Samstag[2], Guido Wabnitz[2], Michael Kirschfink[2], Reinhard Wallich[2], G. Maria Hänsch[2], Inaam A. Nakchbandi[1,2]

Journal of Molecular Medicine 2015

Supplementary Figure 1

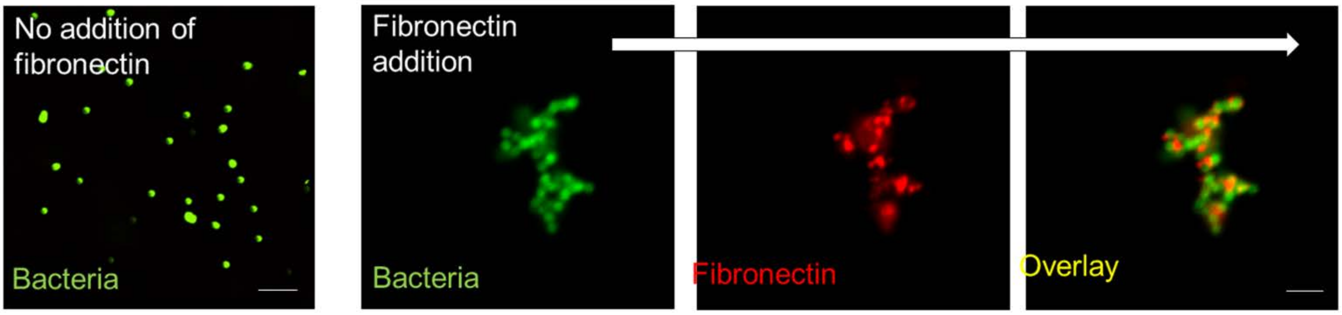

Studies on the effect of exogenous fibronectin addition on *Staphylococcus aureus* of the Wood strain 46.
Fibronectin binds to opsonized bacteria of the Wood strain and results in clumping. Bacteria (in green) were opsonized and then stained for fibronectin. No staining is detected in the absence of exogenous fibronectin (left panel). The remaining three panels show the effect of adding 200 ng/ml fibronectin to the bacteria. Clumping ensues and fibronectin staining reveals its binding to the bacteria. Methods: Opsonized bacteria were left untreated, or were exposed to 200 ng/ml of pFN for 10 minutes, washed and fixed with 4% PFA for 10 minutes. After washing thrice, bacteria were centrifuged and stained for fibronectin as outlined in the methods. *Bars represent 2.5 µm.*
